# Emotion-focused therapy for women with premenstrual dysphoric disorder: a randomized clinical controlled trial

**DOI:** 10.1186/s12888-024-05681-8

**Published:** 2024-07-11

**Authors:** Saeideh Izadi Dehnavi, Seyede Salehe Mortazavi, Mohammad Arash Ramezani, Banafshe Gharraee, Ahmad Ashouri

**Affiliations:** 1 Department of Clinical Psychology, School of Behavioral Sciences and Mental Health, Tehran Institute of Psychiatry, Iran University of Medical Sciences, Tehran, 1445613111 Iran; 2 Geriatric Mental Health Research Center, School of Behavioral Sciences and Mental Health, Tehran Institute of Psychiatry, Iran University of Medical Sciences, Tehran, 1445613111 Iran; 3 Behsa Clinic, Tehran, 1445613111 Iran

**Keywords:** Premenstrual dysphoric disorder, Emotion-focused therapy, Difficulties in emotion regulation, Depression, Anxiety, Stress

## Abstract

**Background:**

Premenstrual dysphoric disorder (PMDD) is a debilitating condition, affecting women of reproductive age. It is characterized by severe periodic physical and psychological symptoms, which end after the onset of menstruation. This study aimed to evaluate the effectiveness of emotion-focused therapy (EFT) for PMDD patients.

**Methods:**

A total of 48 PMDD women, in the age range of 18–44 years, were randomly assigned to two intervention and control groups. The intervention group participated in 16 weeks of EFT treatment, while the control group was selected based on the waiting list (waitlist control group) and followed-up after three months. Forty-four patients finally completed this study. The participants completed the Premenstrual Syndrome Screening Tool (PSST), Difficulties in Emotion Regulation Scale (DERS), and Depression Anxiety Stress Scale-21 (DASS-21) in the first premenstrual period before treatment, the first premenstrual period after treatment, and the premenstrual period three months after treatment.

**Results:**

Based on the repeated measure analysis of variances, the total score of DERS and the total score of PSST decreased significantly (*P* < 0.05). Also, in DASS-21, the scores of depression and stress subscales reduced significantly (*P* < 0.05), while there was no significant decrease in the score of anxiety subscale (*P* > 0.05).

**Conclusion:**

Based on the present results, EFT can be an effective treatment for alleviating the symptoms of PMDD. This treatment can reduce the emotion regulation difficulties of women with PMDD and alleviate the symptoms of depression and stress.

**Trial registration:**

Iranian Registry of Clinical Trials, IRCT ID: IRCT20220920055998N1, Registered on: 12/2/2023.

## Background

Premenstrual dysphoric disorder (PMDD) is a periodic mood disorder, characterized by psychological symptoms (e.g., emotional instability, restlessness, and depressed or anxious mood), cognitive symptoms (e.g., lack of concentration), and physical symptoms (e.g., breast tenderness, feeling bloated, musculoskeletal pain), which frequently occur at the end of the luteal phase of the menstrual cycle [1, 2]. According to statistics, PMDD affects 3–8% of women of reproductive age [3]. The severity of symptoms significantly affects the quality of life of women in the family and society and negatively influences their occupational performance. Evidence suggests that the emotional symptoms of PMDD cause more damage compared to the physical symptoms [4–6]. Since emotional problems constitute a major part of PMDD symptoms, and emotion regulation disorders, depressive symptoms, anxiety, and stress frequently occur in the late luteal phase [7–14], women with PMDD experience more emotional problems compared to healthy people [13, 15]. Additionally, suicidal ideations increase during this period, which is comparable to major depressive disorder [16–18].

Although both pharmacological and non-pharmacological treatments have been proposed for PMDD, most psychological studies have addressed lifestyle modifications [19], including dietary recommendations, exercise [20], and psychological training [21]. Cognitive-behavioral therapies [22] and mindfulness [23] have been also somewhat effective in the treatment of this disorder. However, cognitive-behavioral therapies have mainly focused on the reconstruction of negative cognitive schemas and improvement of coping strategies [24]. Meanwhile, selective serotonin reuptake inhibitors (SSRIs) and contraceptives are the first-line drug treatments for PMDD, which have major side effects despite their effectiveness. Studies have shown that nearly 50% of women who are prescribed SSRIs avoid taking them or stop taking them within six months [25].

Despite the importance of emotions in PMDD, they have not been investigated in the treatment of this disorder. Since many symptoms of women with PMDD are directly or indirectly related to emotions [22–26], a more specialized treatment, which deeply addresses the processes of emotional experience, may be helpful in transforming maladaptive emotions into adaptive ones [27]. Emotion-focused therapy (EFT), which is known as a process-oriented and evidence-based treatment, is a newly introduced therapeutic option [28], introduced by Les Greenberg and colleagues in the 1980’s [29, 30]. This treatment helps the individual identify, experience, explore, and feel their emotions and transform them [28]. In previous research, EFT was found to be effective against disorders, which involve emotions and have common symptoms with PMDD, such as major depressive disorder [31] and generalized anxiety [32]; however, no research has yet addressed the effectiveness of this treatment for PMDD.

In the present study, a 16-session EFT protocol was used for the individual treatment of PMDD. Until now, there has been no individualized treatment based on the EFT approach for this disorder. The current study, which focused on the treatment of PMDD, was conducted on a large sample size and included a control group with a three-month follow-up. Considering the many emotional symptoms of PMDD and its strong impact on women’s lives, it is necessary to focus on reducing emotional pain using new treatments and treatments that focus on emotions such as EFT, in PMDD. We aimed to investigate whether EFT can reduce the severity of PMDD symptoms, depression, anxiety, stress, and difficulties in emotion regulation.

## Methods

### Trial setting and design

The treatment sessions were held during 16 weeks in a random single-blinded manner in an outpatient psychology clinic in Tehran, Iran, from September 23, 2022 to January 20, 2023. Random allocation was performed by one of the researchers who were not involved in the trial. Randomization process was done by Excel 2007 using random block sizes of 4 resulting in 24 participants in each group. Meanwhile, a research assistant, who enrolled the participants and assigned them to the intervention and control groups, was aware of the group assignments. Considering the design of this study, the patients and the therapist were not blinded, while the outcome assessors and analysts were blinded to the assignments. Since there is no specific treatment protocol for PMDD, and there is a common emotional scheme between this disorder and major depressive disorder (both are in the group of mood disorders according to the DSM-5) [1], we used the 16-session EFT protocol for depressive disorders.

### Participants

A total of 78 patients with a diagnosis of PMDD, who were in the age range of 18–44 years, were initially screened using virtual research notifications based on the Premenstrual Syndrome Screening Tool (PSST). For the final diagnosis, in-person interviews were conducted by a clinical expert, using the Structured Clinical Interview for DSM-5 (SCID-5). After obtaining informed consent, the inclusion and exclusion criteria were appraised.

**Fig. 1:**
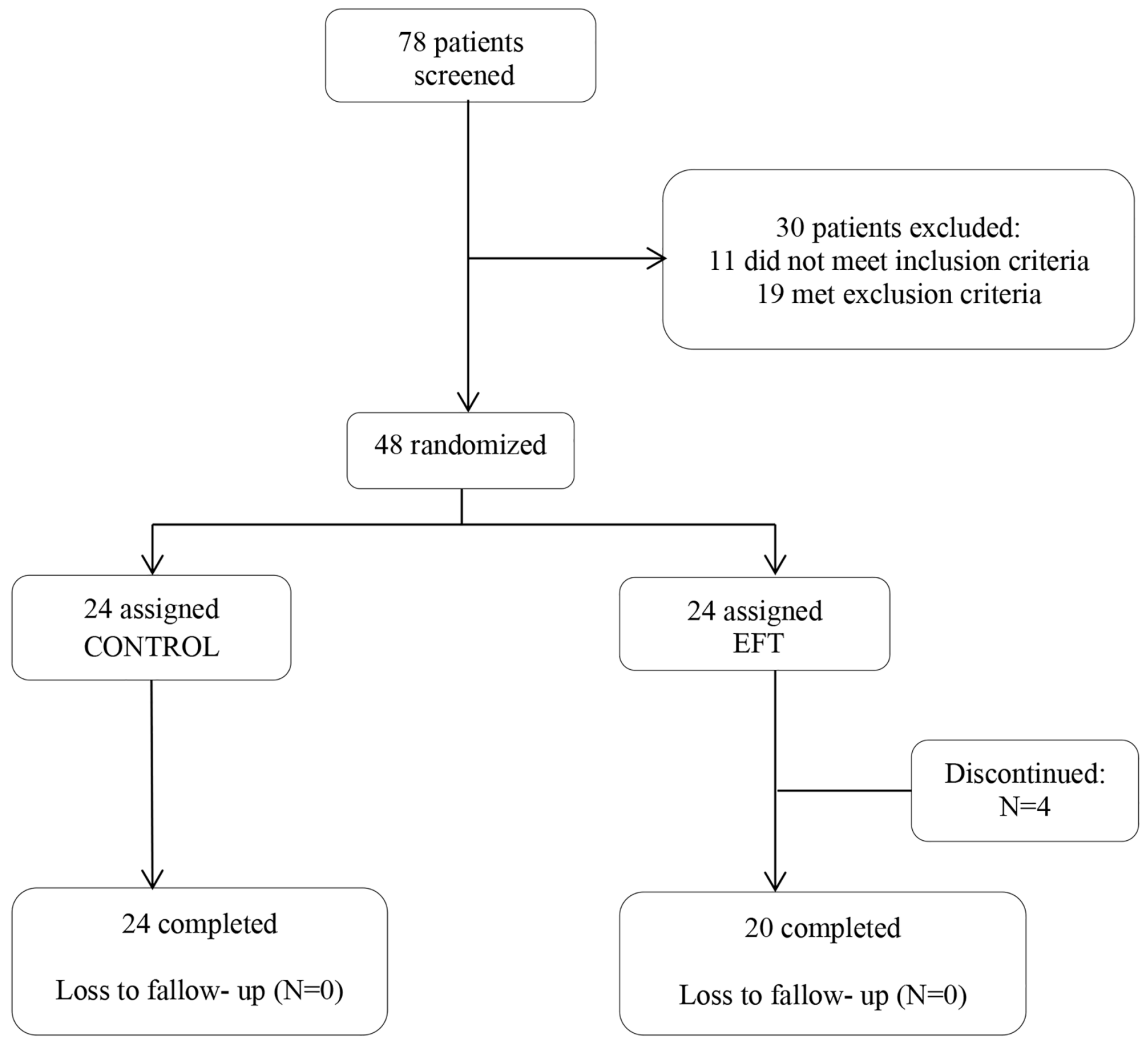
Trial/Participants flow-diagram

The inclusion criteria were being in the age range of 18–44 years, having a regular menstrual cycle, and having a high school diploma at minimum. On the other hand, the exclusion criteria were as follows: pregnancy and breastfeeding in the last six months; having a specific medical disorder (e.g., cardiorespiratory diseases, hypertension, migraine, and hormonal problems) according to self-reports; having a psychiatric disorder for which medications are prescribed; use of hormonal contraceptive methods; and use of addictive drugs. During the trial, the patients were not allowed to receive any psychotherapeutic or pharmacological treatments simultaneously. Finally, 48 patients were randomly selected and included in the intervention. The intervention and control groups each consisted of 24 patients (Table 1). The trial flow diagram and number of dropouts are represented in Fig. 1.

**Table 1:** Demographic characteristic of the participants

Demographic variables		Control group			Intervention group	
		Mean ± SD	Number (%)		Mean ± SD	Number (%)
Age		31.58 ± 4.86			29.33 ± 5.44	
Marital Status	Single		13 (54.2)			15 (62.5)
	Married		11 (45.8)			7 (29.2)
Education	Diploma		0 (0)			1 (4.2)
	Associate		1 (4.2)			3 (12.5)
	Bachelor		7 (29.2)			11 (45.8)
	Master		9 (37.5)			7 (29.2)
	PhD		7 (29.2)			2 (8.3
Job	student		6 (25)			7 (29.2)
	Unemployed		2 (8.3)			8 (33.3)
	housewife		2 (8.3)			1 (4.2)
	Employed		10 (41.7)			6 (25)
	Freelance work		4 (7.16)			2 (8.3)

### Interventions

Eligible participants were randomly assigned to either the intervention or control group for 16 weeks. The EFT was performed based on the treatment plan and interventions proposed by Les Greenberg [31]. The participants completed several self-report scales, including the Premenstrual Syndrome Screening Tool (PSST), Difficulty in Emotion Regulation Questionnaire (DERS), and Depression, Anxiety, and Stress Scale (DASS-21) during the premenstrual period before treatment, the first premenstrual period after treatment, and the premenstrual period three months after treatment. Sessions conducted by PhD student of clinical psychology, trained in EFT, under the supervision of the doctoral-level psychologist and Assistant Professor (With more than 6 years of experience in EFT) of Clinical Psychology. The first treatment sessions included the phases of bonding and emotional awareness; the middle phase involved evoking and exploring maladaptive emotions, and the final phase involved transformation of emotions, as described in Table 2.

Since EFT is an emotion-focused and process-oriented approach, where the patients direct the sessions themselves, the general treatment phases were the same for all the patients; however, each individual was engaged in the treatment at different speeds. The first phase of the intervention focused on creating a safe relationship, along with validating the individual’s feelings in an authentic relationship. Subsequently, the logic of understanding and addressing emotions, besides the importance and types of emotion regulation skills, was discussed, and then, inner emotional experiences were explored to symbolize their meanings and address different markers at different levels of processing. In the next phase, to arouse and stimulate maladaptive emotions, the patient received support so that she could better understand and interact with her emotions [31].

**Table 2:** The content of sessions in the intervention group

Phase	Step
Bonding and awareness	1. Attend to, empathize with, and validate client’s feelings and current sense of self. 2. Provide a rationale for working with emotion. 3. Promote awareness of internal experience. 4. Establish a collaborative focus.
Evocation and exploration	1. Establish support. 2. Evoke and arouse problematic feelings. 3. Undo interruptions. 4. Help client access primary emotions or core maladaptive emotion schemes.
Transformation	1. Help client generate new emotional responses to transform core maladaptive schemes. 2. Promote reflection to make sense of experience. 3. Validate new feelings and support an emerging sense of self.

In this study, we discussed and described the painful experiences of women, using different techniques (e.g., two-chair dialogue and empty-chair technique) to process the core emotion schemes. Next, by eliminating the emerging inhibitions, the therapist helped the patient access her primary emotions. In the final phase, the therapist helped the patient create new emotional reactions for transformation of core maladaptive schemes, promote reflection to give meaning to her experiences and validate new feelings. On the other hand, the intervention for the waitlist control group was performed after the end of treatment and the three-month follow-up.

### Outcome measures

According to the objectives of this study, several tools were used for data collection. The PSST, which contains 19 questions, was the first tool used to collect data. The first 14 questions of PSST evaluate emotional, physical, and behavioral symptoms, while the last five questions evaluate the impact of these symptoms on life. This questionnaire was used for the initial screening of the participants and then, in three phases of the study, that is, before the treatment, after the treatment, and three months after the treatment.

There were three conditions for the initial diagnosis of PMDD: (1) At least one of the responses to items 1–4 is severe; (2) additionally, at least four responses to questions 1–14 are moderate to severe; and (3) at least one of the responses to items related to the impact of symptoms on life is severe [33]. The evaluation of the psychometric properties of the Persian version of this scale, with a Cronbach’s alpha of 0.9, as well as the content validity ratio and content validity index (0.7 and 0.8, respectively), approved the content validity of this questionnaire [6]. The total score of the PSST difference between week 0, week 16, and 3 months follow-up was the primary outcome measure of the trial and difficulties in emotion regulation, depression, anxiety and stress considered as the secondary outcome which were extracted by self-report questionnaires and assessed by three clinical psychologists involved in this research.

In this study, the Structured Clinical Interview for DSM-5 (SCID-5), which is a semi-structured interview for making major DSM-5 diagnoses, was used for the final diagnosis of PMDD. This tool was administered by a trained clinician or a trained mental health expert. It generally takes 45 to 90 min to administer this test [34]. The Persian version of SCID-5 showed a diagnostic sensitivity above 80%. The Kappa coefficient also exceeded 8%, indicating the desirable specificity of this tool [35].

The DERS, containing 36 items [36], was the second questionnaire used in this study for data collection. The Cronbach’s alpha coefficient for the Persian version of this scale was estimated at 0.86 [37].

The final questionnaire was DASS-21, containing seven items for each of the three scales (i.e., depression, anxiety and stress) [38]. Evaluation of the psychometric properties of the Persian version of this scale indicated a Cronbach’s alpha of 0.77 for the depression subscale, 0.79 for the anxiety subscale, and 0.78 for the stress subscale [39].

### Sample size and statistical analyses

G*Power version 3.1.9.2 was used to calculate the sample size. A sample size of 42 was measured for this study. However, considering the attrition rate, a sample size of 48 people was finally selected for the study, and 24 participants were randomly allocated to each of the intervention and control groups. All statistical analyses were conducted in SPSS Version 18. For inferential analysis, repeated measures ANOVA and analysis of covariance (ANCOVA) were used. Studied phases (week 0, week 16 and after 3 months) for each variable were analyzed separately by paired comparisons with Bonferroni correction. A *p*-value of less than 0.05 was considered to be significant.

## Results

To evaluate the equal variance assumption, Shapiro-Wilk test (according to the sample size of the study, < 50) was used, which indicated the normal distribution of data for all variables. Additionally, analysis of variance using Levene’s and Mauchly’s tests confirmed the assumption of sphericity and homogeneity of variance in the data of the EFT and control groups; therefore, we could further analyze the data using the ANOVA method.

The comparison of the intervention and control groups at baseline showed no significant differences in the mean values of the two groups in the pretest. Therefore, the two groups were homogenous regarding the studied variables before the intervention (Table 3). The results of multivariate ANOVA revealed significant interactions between different stages of assessment and also between the intervention and control groups for all the studied variables (except anxiety) (Table 4).

**Table 3:** Main outcome baseline data comparison

Measure (week 0)	Group	Mean	Std. Deviation	*P*-value
DERS	Control	103.70	19.15	0.371
	Intervention	109.16	13.15	
PSST(The severity of the disorder)	Control	38.20	5.99	0.103
	Intervention	40.08	4.63	
Symptoms of PMDD	Control	28.75	4.91	0.276
	Intervention	29.70	3.82	
The effect of symptoms on life	Control	9.45	2.04	0.067
	Intervention	10.37	1.76	
DASS-21 depression	Control	15.50	13.12	0.239
	Intervention	19.58	10.11	
DASS-21 Anxiety	Control	12.25	10.22	0.932
	Intervention	12.08	80.30	
DASS-21 Stress	Control	27.00	8.90	0.721
	Intervention	28.41	7.62	

**Table 4:** Interaction between studied groups (control and intervention) and experiment Phase (week 0, week 16 and after 3 months)

Measure	Phase-Group Interaction (PGI)		
	Wilks’ lambda (F)	*P*-value	Partial Eta Squared
DERS	54.74	0.001	0.73
PSST	61.67	0.001	0.75
DASS-21 Depression	11.90	0.001	0.37
DASS-21 Anxiety	2.39	0.10	0.10
DASS-21 Stress	10.10	0.001	0.33

**Fig. 2:**
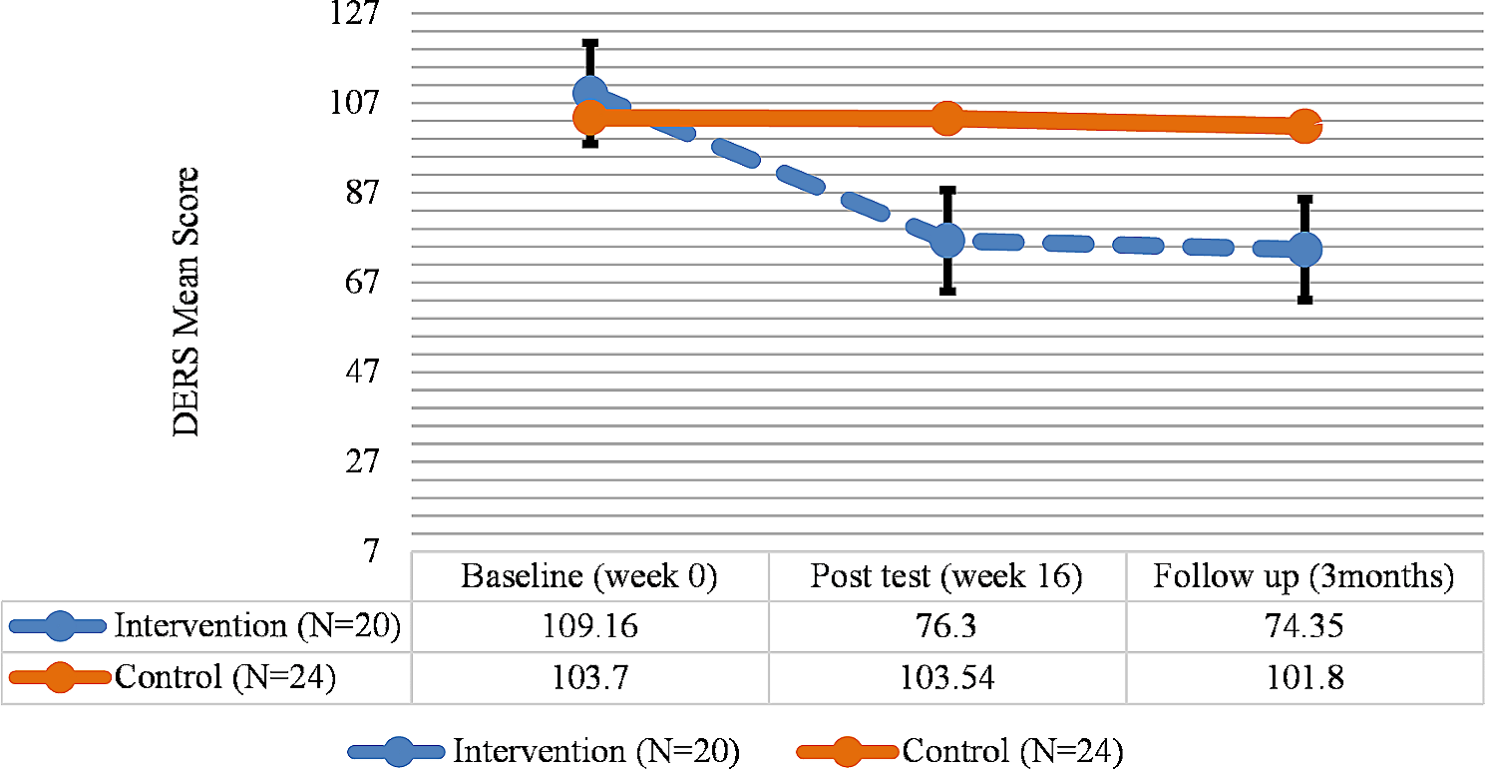
DERS total score trend for each group during the trial

The analysis of the DERS results indicated a significant difference in the emotion regulation difficulties of the intervention group between the pretest and the posttest and follow-up (*P* < 0.05), and a significant reduction was observed in the emotion regulation difficulties. However, in the control group, there was no significant difference between the three phases of assessment (Fig. 2; Table 5).

**Fig. 3:**
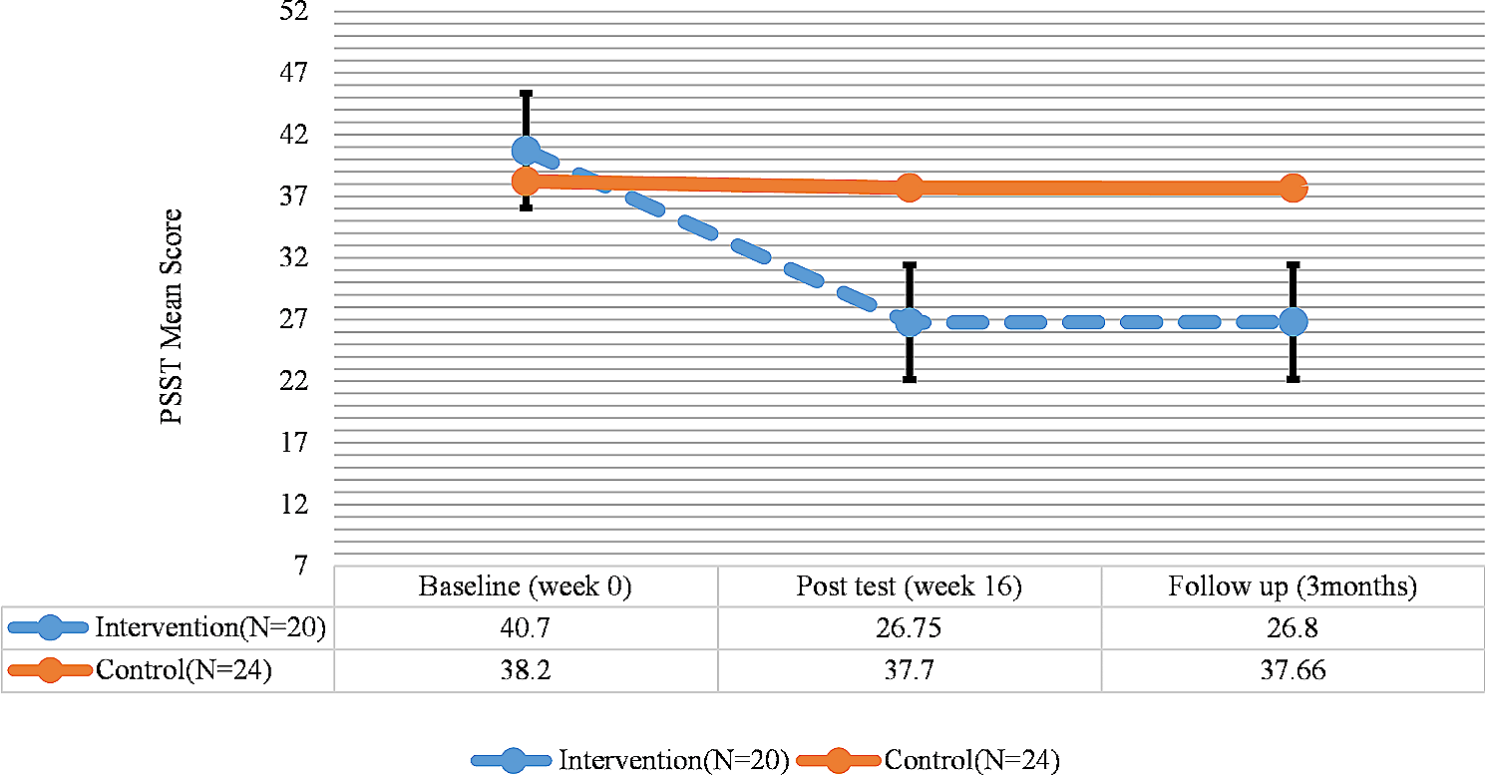
PSST total score trend for each group during the trial

The results of PSST indicated a significant difference in the severity of PMDD symptoms and its components in the intervention group between the pretest and the posttest and follow-up (*P* < 0.05), as the severity of PMDD symptoms and its components significantly diminished; however, no significant difference was observed between the posttest and the follow-up. In the control group, no significant difference was observed in the severity of PMDD symptoms and its components in the pretest, posttest, and follow-up phases (*P* > 0.05) (Fig. 3; Table 5).

**Table 5:** Paired comparisons of studied phase for each variable in both groups

Measure	Groups	Week 0				Week 16	
		Week 16		Follow-Up		Follow-Up	
		Mean Difference	*P*-value	Mean Difference	*P*-value	Means Difference	*P*-value
**DERS**	Control	0.17	1.00	2.6	1.00	2.5	0.99
**PSST**		0.50	1.00	0.54	1.00	0.04	1.00
Symptoms of PMDD		0.38	1.00	0.38	1.00	0.001	1.00
The effect of symptoms on life		0.125	1.00	0.17	1.00	0.04	1.00
**DASS-21 Depression**		-5.92	0.004	-5.09	0.07	0.83	1.00
**DASS-21 Anxiety**		-5.3	0.010	-4.17	0.05	1.17	0.74
**DASS-21 Stress**		-3.42	0.48	-0.92	1.00	2.50	0.60
**DERS**	Intervention	31.95	< 0.001	33.90	< 0.001	0.95	1.00
**PSST**		13.95	< 0.001	13.90	< 0.001	-0.50	1.00
Symptoms of PMDD		9.25	< 0.001	9.10	< 0.001	-0.15	1.00
The effect of symptoms on life		4.70	< 0.001	4.80	< 0.001	0.10	1.00
**DASS-21 Depression**		6.60	0.003	6.90	0.016	0.30	1.00
**DASS-21 Anxiety**		0.30	1.00	0.60	1.00	0.30	1.00
**DASS-21 Stress**		9.40	0.003	10.90	< 0.001	1.50	1.00

**Fig. 4:**
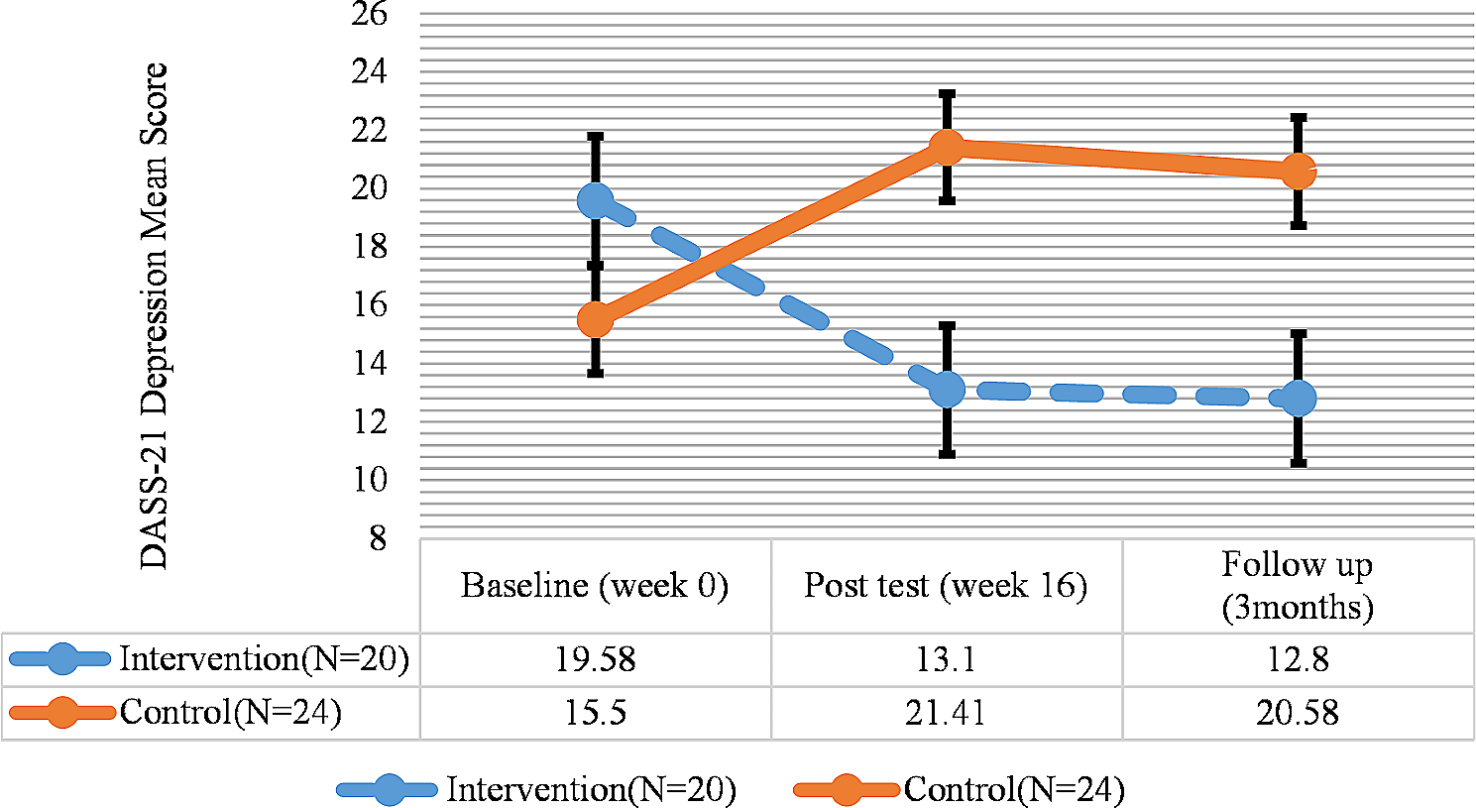
DASS-21 (Depression) total score trend for each group during the trial

Additionally, the results of DASS-21 for depression showed a significant difference in terms of depression in the intervention group between the pretest and the posttest and follow-up (*P* < 0.05), as depression significantly reduced. Nonetheless, no significant difference was observed between the posttest and the follow-up regarding depression. In the control group, there was a significant difference between the pretest and the posttest; the negative score of depression indicated higher depression levels in the control group in the posttest (Fig. 4; Table 5).

**Fig. 5:**
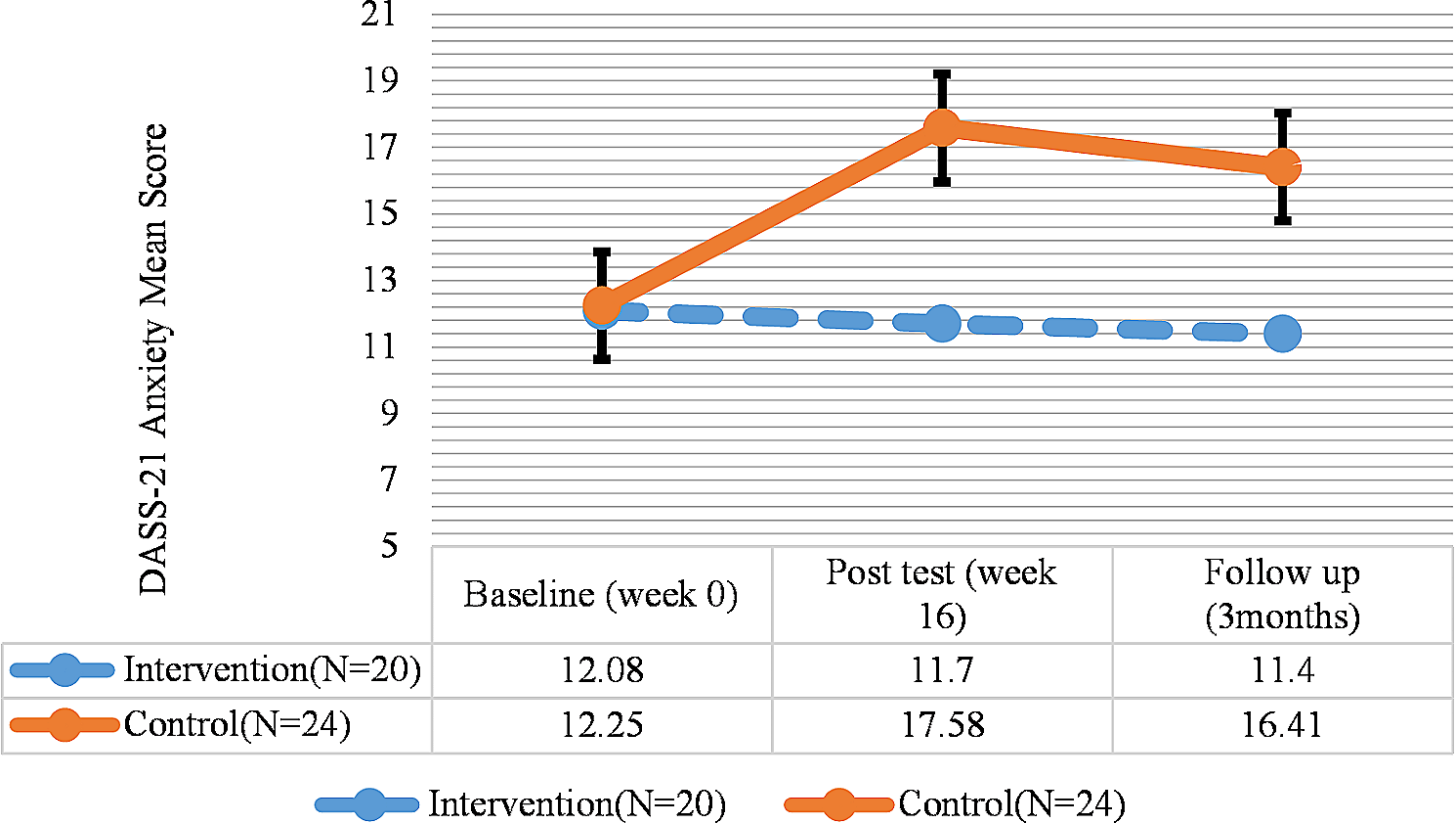
DASS-21(Anxiety) total score trend for each group during the trial

Moreover, the results of DASS-21 for anxiety showed no significant difference in terms of anxiety between the pretest, posttest, and follow-up phases in the intervention group (*P* > 0.05). On the other hand, in the control group, there was a significant difference regarding anxiety between the pretest and the posttest; the negative score of anxiety indicated the higher anxiety levels of the participants in the control group in the posttest (Fig. 5; Table 5).

**Fig. 6:**
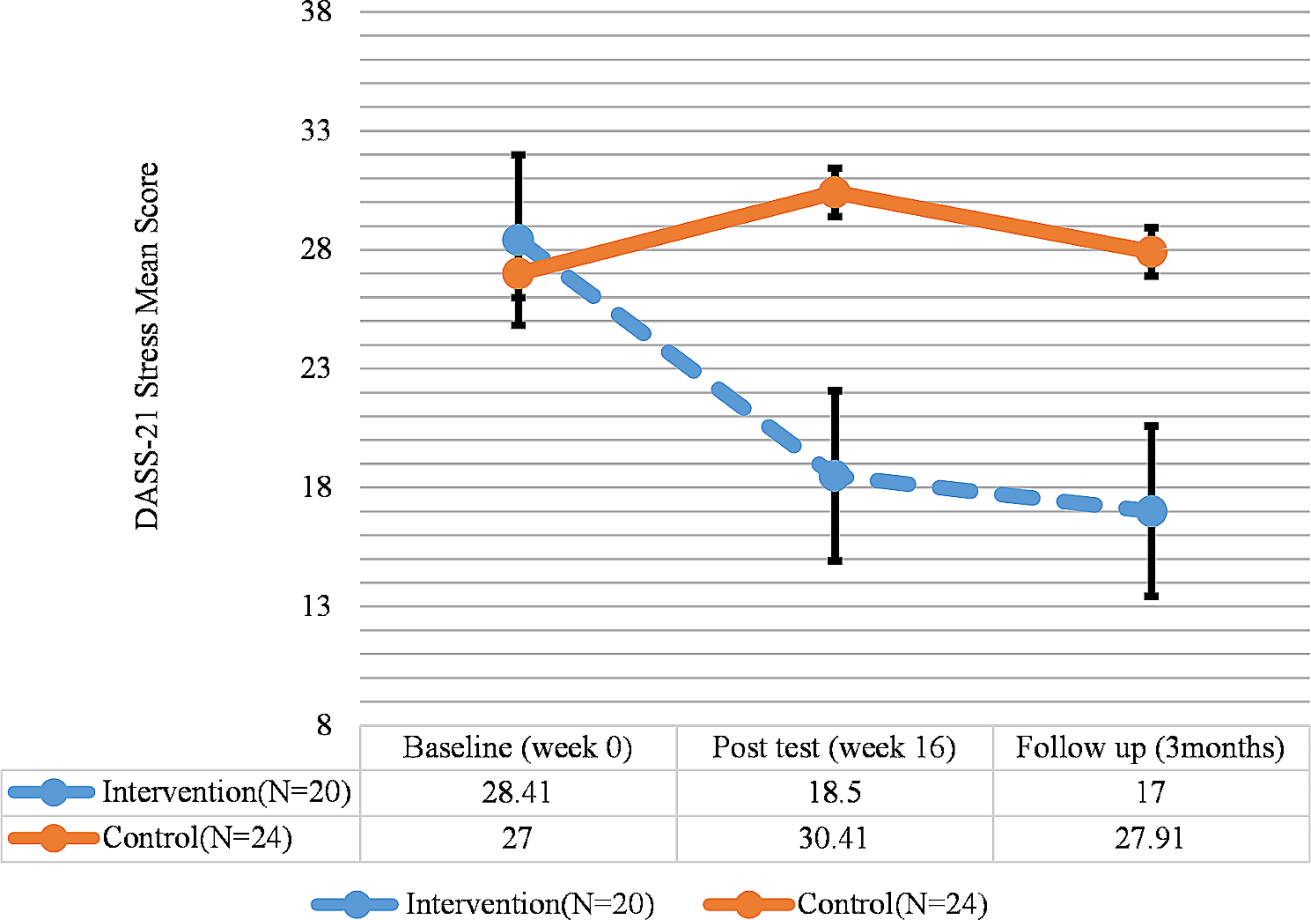
DASS-21(Stress) total score trend for each group during the trial

Finally, the results of DASS-21 for stress indicated a significant difference in terms of stress in the intervention group between the pretest and the posttest and follow-up phases (*P* < 0.05), as a significant decline was observed in stress; however, no significant difference was observed between the posttest and the follow-up regarding stress. On the other hand, there was no significant difference in terms of stress in the control group between the pretest, posttest, and follow-up phases (Fig. 6; Table 5).

## Discussion

The present study aimed to evaluate the effectiveness of EFT for PMDD patients. The results showed that EFT can be a desirable treatment for PMDD. To the best of our knowledge, no study has yet investigated individualized EFT treatments for PMDD. However, since problems in PMDD are majorly emotional [13, 15] and EFT focuses on emotion regulation [40, 41], this treatment could significantly reduce women’s difficulties in regulating their emotions.

Generally, EFT aims to increase emotional intelligence and emotion regulation in a centralized manner. It concentrates on emotional awareness and transparency, control of emotional impulses, and access to emotion regulation strategies [40]. The findings of the present study showed that EFT can be helpful for PMDD patients. Moreover, the present findings indicated the effectiveness of EFT in reducing the severity of PMDD symptoms by alleviating mood, physical, and behavioral symptoms and reducing their negative effects on women’s lives. Research shows that the severity of PMDD symptoms strongly affects women’s quality of life in the family and society and negatively influences their work performance [4, 5, 35]. Based on the present results, EFT could have positive effects on the marital relationship, social life, and family life of women with PMDD in the premenstrual period by reducing the severity of their symptoms. In this regard, a study on group EFT treatment for PMDD reported the alleviation of PMDD symptoms [42]. Other studies have also reported the positive impact of EFT on women’s lives [43].

Since PMDD is placed under the category of mood disorders (according to its symptoms) in DSM-5 [1] and mainly coexists with major depressive disorder [44, 45], the present results indicated the alleviation of depressive symptoms in women with PMDD. Similarly, other studies have reported common emotional schemes and symptoms between PMDD and depressive disorders, which suggest a common treatment for these disorders [14, 46]. On the other hand, SSRIs, which are used against depression, are also used as the first-line psychiatric drugs for PMDD [22]; therefore, the symptoms and treatments of these two disorders are considered similar. Besides, studies focusing on EFT for the treatment of depression have reported the effectiveness of this treatment against depressive disorders [31].

In addition to the coexistence of PMDD with depressive disorders [44], this disorder also coexists with many anxiety disorders [14, 46]. Common medications are used for PMDD and anxiety disorders, which suggests their common physiological overlap and treatment [22]. Meanwhile, previous studies have reported the effectiveness of EFT in the treatment of anxiety disorders [32, 47, 48]. Nevertheless, in the present research, not only there was no significant reduction in the level of anxiety in the intervention group, but also the level of anxiety increased in the control group. Moreover, the level of depression increased in the control group. Considering the concurrence of our research with the social revolution in Iran, which is dominated by women (September 2022) [49], the levels of anxiety and depression have increased in the Iranian society, especially in women [50], which is also evident in our study.

Additionally, the present results indicated a reduction in the levels of stress following EFT for PMDD patients. Generally, high levels of stress are known as a risk factor for PMDD [7, 12]. On the other hand, PMDD is aggravated by stressful life events, and women with severe premenstrual symptoms experience more chronic stress [10]. In primary studies on women with severe premenstrual symptoms, stressors before menstruation were found to be more intense and unpleasant than those of the control group, although the difference was not significant after menstruation.

Hoyer et al. [51] identified higher levels of perceived stress in the luteal phase among women with premenstrual symptoms in the emotional Stroop (ES) tasks. Based on their findings, women with PMDD experienced high stress levels before menstruation. Moreover, retrospective studies indicated that women with PMDD used fewer effective coping strategies, such as rumination and self-focused attention in response to stress [52, 53]. Apparently, EFT, by increasing emotion regulation, can replace other strategies used for stress management. The present results showed that EFT can be a new and promising treatment approach for PMDD, as it can instruct patients to increase their emotional awareness, deepen their affective/emotional experiences, understand their maladaptive emotional responses, and finally, guide their behaviors based on adaptive emotions. Consequently, it leads to a reduction in emotion regulation difficulties, depression, and stress and reduces the severity of PMDD symptoms and their impact on women’s lives.

## Conclusion

As a novel treatment, EFT can help people identify experience, explore, and feel their emotions and transform them. Moreover, EFT is an effective treatment against disorders which involve emotions and have common symptoms with PMDD; therefore, it can be considered a novel promising treatment for mood disorders. Besides, we tried to obtain more detailed information about the symptoms and emotions of women with PMDD by administering questionnaires during the premenstrual period in all three phases of the study. Also, focusing on the transformation of maladaptive emotions into adaptive emotions and the process of reducing maladaptive nuclear pain in the heart of the emotional scheme of this disorder, leads to the reduction of mood and emotional symptoms in PMDD.

### Strengths and limitations

This study had some limitations. First, it was conducted in a single-blinded manner. Second, while the intervention group was receiving treatment, the control group remained inactive. Obviously, delayed treatment of the control group after the follow-up can arouse negative feelings, which may have adverse effects on the final results. It is suggested to conduct similar double-blind studies on a larger and more diverse sample, including a control group, receiving treatment simultaneously with the intervention group. Moreover, it is recommended to evaluate the PMDD symptoms during the premenstrual period and also on other days of the month to obtain more comprehensive results. Also a longer follow-up can contribute to more accurate results. In addition, in this study, the adherence rate was not assess, which should be considered in future studies.

## Data Availability

The dataset supporting the findings of this study cannot be made publicly available for confidentiality and individual privacy reasons. Data are however available from the corresponding author upon reasonable request.

## Abbreviations

DASS-21: Depression, Anxiety, and Stress Scale
DERS: Difficulty in Emotion Regulation Questionnaire
DSM-5: Diagnostic and Statistical Manual of Mental Disorders, 5th edition
EFT: Emotion-Focused Therapy
PMDD: Premenstrual dysphoric disorder
PSST: Premenstrual Syndrome Screening Tool
SCID-5: The Structured Clinical Interview for DSM-5
